# Prediction of whole-body fat percentage and visceral adipose tissue mass from five anthropometric variables

**DOI:** 10.1371/journal.pone.0177175

**Published:** 2017-05-11

**Authors:** Michelle G. Swainson, Alan M. Batterham, Costas Tsakirides, Zoe H. Rutherford, Karen Hind

**Affiliations:** 1 Institute of Sport, Physical Activity and Leisure, Leeds Beckett University, Headingley, Leeds, United Kingdom; 2 Health and Social Care Institute, Teesside University, Middlesbrough, United Kingdom; Universitat de les Illes Balears, SPAIN

## Abstract

**Background:**

The conventional measurement of obesity utilises the body mass index (BMI) criterion. Although there are benefits to this method, there is concern that not all individuals at risk of obesity-associated medical conditions are being identified. Whole-body fat percentage (%FM), and specifically visceral adipose tissue (VAT) mass, are correlated with and potentially implicated in disease trajectories, but are not fully accounted for through BMI evaluation. The aims of this study were (a) to compare five anthropometric predictors of %FM and VAT mass, and (b) to explore new cut-points for the best of these predictors to improve the characterisation of obesity.

**Methods:**

BMI, waist circumference (WC), waist-to-hip ratio (WHR), waist-to-height ratio (WHtR) and waist/height^0.5^ (WHT.5R) were measured and calculated for 81 adults (40 women, 41 men; mean (SD) age: 38.4 (17.5) years; 94% Caucasian). Total body dual energy X-ray absorptiometry with Corescan (GE Lunar iDXA, Encore version 15.0) was also performed to quantify %FM and VAT mass. Linear regression analysis, stratified by sex, was applied to predict both %FM and VAT mass for each anthropometric variable. Within each sex, we used information theoretic methods (Akaike Information Criterion; AIC) to compare models. For the best anthropometric predictor, we derived tentative cut-points for classifying individuals as obese (>25% FM for men or >35% FM for women, or > highest tertile for VAT mass).

**Results:**

The best predictor of both %FM and VAT mass in men and women was WHtR. Derived cut-points for predicting whole body obesity were 0.53 in men and 0.54 in women. The cut-point for predicting visceral obesity was 0.59 in both sexes.

**Conclusions:**

In the absence of more objective measures of central obesity and adiposity, WHtR is a suitable proxy measure in both women and men. The proposed DXA-%FM and VAT mass cut-offs require validation in larger studies, but offer potential for improvement of obesity characterisation and the identification of individuals who would most benefit from therapeutic intervention.

## Introduction

In clinical practice, public health and the wider health and fitness industry, obesity is conventionally defined using the body mass index (BMI) criterion, with a value of ≥ 30 kg/m^2^ categorising both men and women as obese [1]. Although there are many benefits to using BMI, particularly for population-based screening, evidence indicates the existence of obesity sub-groups such as metabolically healthy but obese, or metabolically unhealthy but normal weight [2, 3]. Therefore, use of BMI only, is potentially falling short of identifying those at an increased risk of associated conditions, in particular cardio-metabolic diseases. Strong associations have been identified between whole body and regional fat mass with risk of certain diseases [4], and as such research indicates that visceral adiposity is an independent predictor of all-cause mortality in men and women [5, 6]. More recently, greater emphasis has been placed upon the relationships between visceral adiposity within the abdominal region (visceral adipose tissue; VAT) and components of metabolic syndrome [7], insulin resistance [8, 9], cardiovascular disease [10, 11] and even non-spine fractures [12], indicating that VAT is an important target for investigation.

VAT can be quantified with confidence using imaging modalities such as abdominal computed tomography (CT). However, due to high radiation exposure and cost implications, CT is not ideal for large-scale screening or routine practice. However, recent advancements in densitometry imaging have led to a new Corescan software that enables the quantification of VAT through total body dual-energy x-ray absorptiometry (DXA) scans, with significantly lower radiation and expense than CT [13–15]. Although confounded by levels of subcutaneous fat [10], proxy measures of fat mass, such as BMI, waist circumference (WC) and waist-to-hip ratio (WHR) are more frequently used [16]. In particular, there is an emerging appreciation of waist-to-height ratio (WHtR) as a risk indicator for poor cardio-metabolic health [17–23]. Ashwell [24, 25] first devised the WHtR, proposing that cut-point values of 0.5 and 0.6 can identify individuals who are at increased health risk and substantial health risk, respectively. It is important to evidence the validity of proxy measures of overall and visceral obesity using gold standard criteria, which would support their use in practice. Roriz et al. [26] performed CT scans in 191 adults and this is the only study to date that has provided evidence supporting the notion that WHtR is a good predictor of visceral obesity (defined as >130cm^2^). These authors proposed WHtR cut-points in 20–59 year olds of 0.54 in men and 0.59 in women, and 0.55 (men) and 0.58 (women) in participants ≥60 years. Recently, a new index was proposed—waist/height^0.5^—that was a stronger predictor of cardiometabolic risk than other anthropometric variables, including WHtR, BMI, WC, a body shape index, and WHR [27].

There is also growing interest in the clinical utility of multiple anthropometric measures for identifying individuals at risk of cardiometabolic diseases, but this could be time-consuming, and not always feasible or realistic in practice. Research indicates that compared to separate measures, BMI and WC combined can more accurately predict abdominal fat mass [10]. Similarly, BMI and WHtR combined, can identify elevated cardiovascular disease risk better than BMI alone [28]. There have been several studies that have used DXA-derived VAT, although these have mainly reported on the ability of DXA-VAT to predict cardiometabolic risk and not its utility for obesity identification [29, 30].

The current study measured VAT mass and overall fat mass as a percentage of body mass (%FM) using DXA imaging in UK men and women. The main aims were (a) to compare 5 separate anthropometric variables in the prediction of %FM and DXA-VAT mass and (b) to identify tentative new obesity cut-off points for the anthropometric variable in the model found to be the best from the five candidates.

## Materials and methods

### Participants and ethics approval

As part of a University-wide health screening programme, data from eighty-one adults were analysed which included a heterogeneous sample of 41 men (mean (SD) age: 40.5 (20) years; BMI: 26.3 (4.1) kg/m^2^) and 40 women (age: 36.3 (14.5) years; BMI: 24.8 (4.4) kg/m^2^). The ethnicity distribution was 94% Caucasian (n = 76), 5% Indian/Pakistani (male n = 2; female n = 2), and 1% African-Caribbean (male n = 1). The study was reviewed and approved by the Leeds Beckett University Research Ethics Committee, and in accordance with the Declaration of Helsinki. All participants provided signed informed consent to participate in the study, and all data was collected between 08:00 am and 12:00 noon during a single visit to the laboratory.

### Anthropometric measurements

Participants were measured wearing light loose clothing or a hospital gown, and no jewellery or footwear. Body mass was measured using calibrated, digital flat platform scales (Seca Alpha, SECA, Birmingham, UK) to the nearest 0.1 kg. Standing height was measured to the nearest 0.1 cm using a free-standing stadiometer (SECA, Birmingham, UK) and body mass index (BMI) was subsequently calculated using the standard Quetelet formula (mass divided by squared height) (kg/m^2^) with categories in accordance with the WHO [1] guidelines, more specifically obesity defined by a BMI ≥30 kg/m^2^. Waist circumference (WC) was measured at the midway point between the iliac crest and the lowest rib [31] to the nearest 0.1 cm. Hip circumference (HC) was measured at the widest part of the buttocks [31] to the nearest 0.1 cm in order to calculate waist-to-hip ratio (WHR) by the simple division of WC/HC. Subsequently, waist to height ratio (WHtR), a more contemporary measure, was calculated by WC/Height [32]. We also calculated an index proposed recently as a superior predictor of cardiometabolic risk—WC/Height^0.5^ (WHT.5R; [27]).

### DXA-derived measurements

Each participant received one total body fan-beam dual energy X-ray absorptiometry (GE Lunar iDXA, GE Healthcare, Madison, WI) scan, from which body composition, namely percentage fat mass (%FM), was determined. VAT was quantified using the validated CoreScan software (EnCore version 15.0). Participants were placed in the supine position on the scanning table and the body aligned with the central horizontal axis. Arms were positioned parallel to, but not touching the body, with a 1 cm space in between the thigh and the hand. Forearms were pronated with hands flat on the bed. Legs were fully extended and feet were secured with a canvas and Velcro support to avoid foot movement during the scan acquisition. Scans were conducted using standard (153 mm/sec) or thick (80 mm/sec) mode depending on body stature. One skilled DXA technologist led all scans and analyses, which were checked by an International Society for Clinical Densitometry (ISCD) clinically certified densitometrist. The regions of interest (ROI) for the total body cut-offs were manually adjusted according to the manufacturer’s instructions. The ROI over the android region for the assessment of VAT was automated by the software. Precision error for our Unit has previously been published for both fat mass [33] and VAT mass [15]. The machine’s calibration was checked and passed on a daily basis using the GE Lunar calibration hydroxyapatite and epoxy resin phantom. There was no significant drift in calibration for the study period.

### Data analysis

Linear regression analyses were used to compare five candidate models in the prediction of whole body fat percentage and VAT mass. The five anthropometric predictors compared were BMI, WC, WHR, WHtR, and WHT.5R. Diagnostic plots revealed badly behaved residuals for the VAT models; therefore, VAT was log-transformed prior to the primary analysis. All analyses were stratified by sex. We used an information-theoretic approach (Akaike’s Information Criterion; AIC) to compare the five candidate models, separately for both whole body fat percentage and VAT [34]. The model with the lowest AIC identifies the best of the candidate models, and provides a reference for model comparison. The remaining models are then compared to the best model and evaluated using the difference in AIC (AICΔ) according to the following scale [35]: Essentially equivalent model (AICΔ <2), plausible alternative (AICΔ 2–7), weak support (AICΔ 7–14), and unsupported (AICΔ >14). The AIC identifies the best model of a set of candidates, but its meaningfulness depends on there being a good predictive model in the set. Therefore, we also present adjusted R^2^ and the standard error of the estimate for all models.

Following the identification of the best model, we used the prediction equation to derive tentative cut-points for identifying obesity based on %FM and VAT mass. For the %FM equation, standard thresholds of 25% and 35% were used as the definition of obesity in men and women respectively. However, due to no known cut-points for VAT mass derived by DXA in males or females, we generated distributional tertiles whereby the highest third was proposed as the obese group. For both %FM and VAT mass, we derived the cut-point for the best anthropometric predictor that resulted in a probability of ≥0.75 (odds of 3:1 in favour) of an individual being obese, as defined above, given the prediction error from the regression equation (standard error of the estimate; SEE). To account for the downward bias of the SEE associated with small sample sizes (<50) for men and women groups, we adjusted the SEE upwards required in the derivation of the cut-points. A probability of ≥0.75 was selected as this is the threshold denoting “likely to be” in the magnitude-based inferences framework [36]. Briefly, the probability that an individual’s true %FM or VAT mass value is greater than some threshold value—given the predicted value from a regression equation—is obtained from the one-tailed area under the t-distribution for the appropriate degrees of freedom at the following t value:

t=(Predicted value minus threshold value for obesity)/standard error of the estimate.

In small validity studies (n<50), due to sampling variability in the prediction equation, the standard error of the estimate should ideally be adjusted upwards by a factor given by: $\sqrt{\left(1+1/\text{n}+1/\left(\text{n}-3\right)\right)}$. Knowing the t-value associated with a probability of 0.75, the threshold value for obesity, and the standard error of the estimate allows us to derive the required predicted value. We can then derive the cut-point for the anthropometric predictor by rearranging the obtained regression equation. A worked example is provided in the Results. All analyses were conducted using SPSS Statistics software (v.23, Armonk, NY: IBM Corp).

## Results

Descriptive data for the five anthropometric predictors and the two dependent variables (%FM and VAT mass) are shown in Table 1.

**Table 1:** Sample characteristics.

Obesity measure	Men (n = 41)	Women (n = 40)
BMI (kg/m^2^)	26.2 (4.1)	24.8 (4.4)
BMI ≥30kg/m^2^	17% (n = 7)	10% (n = 4)
WC (cm)	88.8 (12.3)	78.7 (13.0)
WHR	0.91 (0.07)	0.80 (0.07)
WHtR	0.50 (0.07)	0.48 (0.08)
WHT.5R	0.67 (0.09)	0.61 (0.10)
Total body fat mass (%)	25.5 (8.4)	34.0 (7.8)
FM >25%	54% (n = 22)	-
FM >35%	-	48% (n = 19)
VAT mass (g)	604 ×/÷ 2.9	204 ×/÷ 4.9

Data presented as Mean (SD)

BMI—Body mass index; WC—waist circumference; WHR—waist-to-hip ratio; WHtR—waist-to-height ratio; WHT.5R –WC/height^0.5^; fat mass (FM); visceral adipose tissue (VAT).

For VAT mass, which was log-transformed prior to analysis, the geometric mean is shown, with the dispersion given as a ×/÷ factor standard deviation (SD) [36].

Only around 1 in 6 men and 1 in 10 women were obese according to the BMI criterion versus around half of each sample according to the whole-body fat percentage thresholds. The comparison of candidate models for the prediction of %FM (Table 2) and VAT mass (Table 3) revealed that the WHtR was the best predictor in both men and women.

**Table 2:** Prediction of whole body fat percentage from anthropometric measures.

	AIC Difference (Inference)	Adjusted R^2^	Standard Error of Estimate*
**Males** (n = 41)			
BMI	8 (weak support)	0.71	4.5
WC	5 (plausible)	0.73	4.4
WHR	50 (unsupported)	0.19	7.5
WHtR	0 (best	0.76	4.1
WHT.5R	<1 (equivalent)	0.76	4.1
**Females** (n = 40)			
BMI	8 (weak support)	0.51	5.5
WC	6 (plausible)	0.53	5.3
WHR	27 (unsupported)	0.21	6.9
WHtR	0 (best)	0.60	5.0
WHT.5R	2 (plausible)	0.57	5.1

AIC—Akaike’s Information Criterion; BMI—Body mass index; WC—waist circumference; WHR—waist-to-hip ratio; WHtR—waist-to-height ratio; WHT.5R –WC/height^0.5^

*The 95% confidence interval for the standard error of the estimate is ×/÷ a factor of 1.25 at these degrees of freedom.

**Table 3:** Prediction of VAT mass (Log) from anthropometric measures.

	AIC Difference (Inference)	Adjusted R^2^	Standard Error of the Estimate ×/÷ factor (95% CI)
**Males** (n = 41)			
BMI	13 (weak support)	0.60	2.0 (1.8 to 2.4)
WC	4 (plausible)	0.68	1.8 (1.6 to 2.1)
WHR	41 (unsupported)	0.21	2.6 (2.2 to 3.3)
WHtR	0 (best	0.71	1.8 (1.6 to 2.1)
WHT.5R	<1 (equivalent)	0.71	1.8 (1.6 to 2.1)
**Females** (n = 32*)			
BMI	6 (plausible)	0.58	2.8 (2.3 to 4.1)
WC	3 (plausible)	0.61	2.7 (2.2 to 3.7)
WHR	25 (unsupported)	0.22	4.1 (3.0 to 6.7)
WHtR	0 (best)	0.65	2.6 (2.2 to 3.7)
WHT.5R	<1 (equivalent)	0.64	2.6 (2.2 to 3.7)

AIC—Akaike’s Information Criterion; BMI—Body mass index; WC—waist circumference; WHR—waist-to-hip ratio; WHtR—waist-to-height ratio; WHT.5R - WC/height^0.5^

*8 females excluded due to undetectable levels of VAT mass leading to badly behaved residuals

The individual cut-point for WHtR associated with a probability of being obese (>25% fat in men and >35% fat in women) of ≥0.75 was 0.53 in men and 0.54 in women. Below, as an illustration of the method, we present the derivation of the male cut-point. The value from the t-distribution associated with a probability of 0.75 at 39 degrees of freedom (n-2) is 0.681. The standard error of the estimate from the prediction equation was 4.1% fat (adjusted to 4.2%). From the Methods:

t=(Predicted value minus threshold value for obesity)/standard error of the estimate.

Therefore, the required predicted value is given by:

Predicted=25+4.2×0.681=27.9.

The derived prediction equation was: %FM = 99.7 × WHtR– 24.7. Therefore, the required WHtR cut-point is given by: (27.9+24.7)/99.7 = 0.53.

The individual cut-point for WHtR associated with a probability ≥0.75 of being in the highest third for VAT mass (>1108 g for men and > 477 g for women) was 0.59 in both men and women.

## Discussion

Our main finding is that from five anthropometric variables (BMI, WC and WHR, WHtR, and WHT.5R) the WHtR is the best predictor of DXA-derived whole body fat percentage and VAT mass, in both men and women. The new WHT.5R index was an essentially equivalent predictor of VAT mass in both sexes. For whole body fat percentage, WHT.5R was an equivalent predictor in males and a plausible alternative in females. Waist circumference (unadjusted for height) was a plausible alternative model for both sexes for both outcomes. Models with BMI as the predictor had weak support for the prediction of whole body fat percentage in both sexes and for VAT mass in males. However, BMI was a plausible alternative for the prediction of VAT mass in females. The use of WHR was unsupported in all models. Even for the best model, the R^2^ and standard errors of the estimate reveal that simple anthropometric indices are uncertain predictors of DXA-measured body composition outcomes, with prediction errors similar to, if slightly larger, than those of skinfold methods [37, 38].

It is encouraging that WHtR was identified as the best predictor of both whole-body fat percentage and VAT mass in both men and women, whereas to account for both factors, a clinician would typically need to measure both BMI and WC, which require differential categorisation by age, sex and ethnicity that is not required for WHtR [39]. In contrast, in NHANES survey participants, Heo et al. [40] investigated optimal scaling for both weight and WC to height in the prediction of DXA-measured total %FM, and reported that WC alone, without adjustment for height, is the optimal index for both sexes. However, in the current study we found that WHtR was the best model, although WC was still a plausible alternative. Indeed, WC alone, unadjusted for height, might be more appealing in the clinical setting as it requires just a single measurement. These findings highlight that these simple measurements may be used as surrogates, reducing the need for DXA scans in the clinical setting.

In the current study, the AIC differences (Tables 2 and 3) revealed that models with WHR as the predictor were by far the worst of all candidate models, and were unsupported. The rationale supporting the use of WHR is that it accounts for central and peripheral fat distribution [31], but changes in WC and hip circumference are relative to each other so favourable weight loss will not necessarily lead to reductions in WHR. This observation would therefore support that WHR is not ideal for identification of obesity or monitoring changes in weight status, hence it is not included in UK National Institute for Health and Care Excellence guidance [41]. Our study indicates that monitoring WC in absolute terms or in relation to the practically unchanged anthropometric measure of height is a better surrogate measure for levels of adiposity with the propensity to cause cardiometabolic diseases.

Despite the generally well-accepted understanding that BMI has its limitations, it is still valuable for population-level screening with Ortega and colleagues [42] recently reporting that that BMI was a better predictor of mortality than fat mass, when determined by hydrostatic weighing or skinfold thickness, in 60,000 adults. Also, WC—a plausible alternative to WHtR in the current study—is generally accepted as a proxy for central adiposity, especially as there are well-established risk categories and it is recommended in the UK National Institute for Health and Care Excellence 2014 guidelines for obesity assessment [41]. Despite this recommendation and the growing evidence-base in support of waist measurements, emphasis from NICE remains on weight and BMI with focus on WC only in those with a BMI under 35 kg/m^2^. In the current study, around 1 in 7 participants were classified as obese using the conventional BMI criterion versus 1 in 2 participants using whole-body fat percentage criteria. This observation suggests that sole use of BMI may be misleading. The finding is underscored by the fact that models with BMI as the predictor of whole-body fat percentage had weak support in both sexes.

A second major outcome of our study was identification of obesity cut-off points for WHtR based on percentage fat mass and VAT mass. The derived cut-points for whole-body fat percentage were almost identical in men (0.53) and women (0.54). Moreover, our study is the first to derive a tentative cut-point for WHtR in the prediction of high DXA-derived VAT mass (defined as the highest third in our sample distribution, in the absence of an established threshold). Remarkably, in a cross-sectional study of 191 adults, Roriz et al. [26] reported WHtR cut-points ranging from 0.54 to 0.59 for predicting high visceral adiposity (VAT area of ≥ 130 cm^2^ determined by CT) in men and women aged 20–59 years and ≥ 60 years, respectively. Elsewhere, it has been reported that WHtR is a proxy for visceral adiposity and the existing cut-points of >0.5 and >0.6 are related to high and very high health risk (obesity and metabolic syndrome) respectively [43]. Our study differs as we propose cut-off points for visceral adiposity defined arbitrarily as the upper third of the distribution. Indeed, our proposed 0.59 cut-point aligns closely to Ashwell's 0.6, which is often referred to as the threshold above which patients should be advised to “take action” based on the knowledge of visceral fat-associated links with negative health outcomes [5–7, 10, 11]. Our findings also support Ashwell and Gibson [23] in their proposal that WHtR should be fully considered as a replacement for the BMI and WC combined matrix, which could support obesity characterisation when DXA is not available or not desirable.

Although it is accepted that use of DXA is not feasible in all clinical settings, for those who do have access we also highlight the advantages of using DXA to obtain important and precise information on fat mass and VAT [15, 33], which as an adjunct to WHtR, might further improve identification of patients at risk of associated negative health outcomes. A prospective study on a larger scale would enable evaluation of this hypothesis. In this way, an individual participant exceeding a valid and robust WHtR cut-point could perhaps be used as a simple clinical indicator for further exploration with more sophisticated methods. This form of risk stratification would require more research identifying the WHtR cut-points that best ‘rule in’ and ‘rule out’ negative health outcomes.

It is well-documented that the number of adults who are defined as obese differs depending on the measurement method used [44]. Relying, therefore, on just one standard measure of obesity may lack the required accuracy for the identification of individuals with adiposity at risk of adverse health outcomes. In an effort to promote the simple public health message of “keep your waist less than half of your height”, it was recently reported that 10% of the UK population would be misclassified if only BMI is used, and over one quarter of people with a healthy BMI (18.5–24.9 kg/m^2^) are misclassified when WHtR >0.5 is implemented [39]. Our results indicate that using BMI alone classified a much lower number of participants as obese, in comparison to obesity cut-points for measures of WHtR and %FM; this observation cannot be confirmed for visceral obesity due to the arbitrary nature of our VAT mass classification. Even in a small sample, this is somewhat alarming and does provide further evidence that alternative measures are fundamental to the more accurate identification of obesity, therefore ensuring that individuals are referred to the most suitable therapeutic approach to reduce risk of obesity-related conditions.

Strengths of our study include the use of precise DXA measures of body composition outcomes, the use of information-theoretic methods for robust model comparison, and the application of a novel method to derive cut-points for predicting obesity status. However, it is important to acknowledge explicitly some key limitations. First, the sample sizes of men and women are small for a validity study. We have presented cut-points based on standard errors of the estimate adjusted for sample size (inflated prediction error resulting from sampling variability) but, nonetheless, our findings need to be replicated in a large definitive measurement study. Second, our sample is 94% Caucasian, and our findings cannot be generalised to other ethnic groups.

### Research and practical implications

Our findings indicate that WHR should not be relied on in clinical practice for obesity identification. Interestingly, however, in middle-to-older aged adults WHR has been shown to have a greater predictive ability to identify health outcomes than %FM and BMI [45]. Our study has devised tentative new cut-off points that need to be validated in a larger sample and could potentially be utilised in further research and in clinical practice. One key strength of our identified cut-points is that, despite using different equations for men and women, the generated threshold values are virtually identical. If replicated, this provides a consistent and simple message to clinicians that can be transferrable to the general public. The cut-points are more specific but still align broadly to the WHtR guidance that adults and children should keep WC to less than half their height i.e. <0.5 [39, 46]. A further advantage of using WHtR is cost effectiveness given that this measure only requires the use of a tape measure and stadiometer, both of which are inexpensive and portable. We advise that future prospective research should implement and test these new cut-points to explore associations with negative health outcomes such as cardiometabolic disease, especially with stratification by obesity phenotypes of metabolically healthy but obese, or metabolically unhealthy but normal weight, in an effort to increase capture of at-risk individuals.

In conclusion, our data indicate that in this study WHtR is the best predictor of the five models compared for obesity characterisation in adults, and in combination with DXA-derived fat mass and/or VAT mass, the proposed cut-off points might improve obesity identification in both men and women. Our results cannot yet be generalised to other population groups but provide new information which is intended to provide direction for validation and/or exploration in future studies.

## Supporting information

S1 File Swainson et al—PloSOne final data set. (SAV)

S1 Table Sample characteristics. Data presented as Mean (SD) BMI—Body mass index; WC—waist circumference; WHR—waist-to-hip ratio; WHtR—waist-to-height ratio; WHT.5R –WC/height^0.5^; fat mass (FM); visceral adipose tissue (VAT). For VAT mass, which was log-transformed prior to analysis, the geometric mean is shown, with the dispersion given as a ×/÷ factor standard deviation (SD) [36].
(DOCX)

S2 Table Prediction of whole body fat percentage from anthropometric measures. *AIC—Akaike’s Information Criterion; BMI—Body mass index; WC—waist circumference; WHR—waist-to-hip ratio; WHtR—waist-to-height ratio;* WHT.5R –WC/height^0.5^ *The 95% confidence interval for the standard error of the estimate is ×/÷ a factor of 1.25 at these degrees of freedom.
(DOCX)

S3 Table Prediction of VAT mass (Log) from anthropometric measures. *AIC—Akaike’s Information Criterion; BMI—Body mass index; WC—waist circumference; WHR—waist-to-hip ratio; WHtR—waist-to-height ratio;* WHT.5R - WC/height^0.5^ *8 females excluded due to undetectable levels of VAT mass leading to badly behaved residuals.
(DOCX)
