# Supplementary material for: Prediction of whole-body fat percentage and visceral adipose tissue mass from five anthropometric variables
Source: PLoS One. 2017 May 11;12(5):e0177175. doi: 10.1371/journal.pone.0177175 (PMC5426673; doi:10.1371/journal.pone.0177175)
Supplement: S1 Table — Data presented as Mean (SD) BMI—Body mass index; WC—waist circumference; WHR—waist-to-hip ratio; WHtR—waist-to-height ratio; WHT.5R –WC/height0.5; fat mass (FM); visceral adipose tissue (VAT). For VAT mass, which was log-transformed prior to analysis, the geometric mean is shown, with the dispersion given as a ×/÷ factor standard deviation (SD) [36]. (DOCX) [file pone.0177175.s002.docx]

**Table 1: Sample characteristics**

| Obesity measure | Men  (n=41) | Women  (n=40) |
| --- | --- | --- |
| BMI (kg/m^2^) | 26.2 (4.1) | 24.8 (4.4) |
| BMI ≥30kg/m^2^ | 17% (n=7) | 10% (n=4) |
| WC (cm) | 88.8 (12.3) | 78.7 (13.0) |
| WHR | 0.91 (0.07) | 0.80 (0.07) |
| WHtR | 0.50 (0.07) | 0.48 (0.08) |
| WHT.5R | 0.67 (0.09) | 0.61 (0.10) |
| Total body fat mass (%) | 25.5 (8.4) | 34.0 (7.8) |
| FM >25% | 54% (n=22) | - |
| FM >35% | - | 48% (n=19) |
| VAT mass (g) | 604 ×/÷ 2.9 | 204 ×/÷ 4.9 |

Data presented as Mean (SD)

BMI - Body mass index; WC – waist circumference; WHR – waist-to-hip ratio; WHtR – waist-to-height ratio; WHT.5R – WC/height^0.5^; fat mass (FM); visceral adipose tissue (VAT).

For VAT mass, which was log-transformed prior to analysis, the geometric mean is shown, with the dispersion given as a ×/÷ factor standard deviation (SD) [36].
