# Supplementary material for: Prediction of whole-body fat percentage and visceral adipose tissue mass from five anthropometric variables
Source: PLoS One. 2017 May 11;12(5):e0177175. doi: 10.1371/journal.pone.0177175 (PMC5426673; doi:10.1371/journal.pone.0177175)
Supplement: S2 Table — AIC—Akaike’s Information Criterion; BMI—Body mass index; WC—waist circumference; WHR—waist-to-hip ratio; WHtR—waist-to-height ratio; WHT.5R –WC/height0.5 *The 95% confidence interval for the standard error of the estimate is ×/÷ a factor of 1.25 at these degrees of freedom. (DOCX) [file pone.0177175.s003.docx]

**Table 2: Prediction of whole body fat percentage from anthropometric measures**

|  | AIC Difference  (Inference) | Adjusted R^2^ | Standard Error of Estimate* |
| --- | --- | --- | --- |
| **Males** (n=41) |  |  |  |
| BMI | 8 (weak support) | 0.71 | 4.5 |
| WC | 5 (plausible) | 0.73 | 4.4 |
| WHR | 50 (unsupported) | 0.19 | 7.5 |
| WHtR | 0 (best | 0.76 | 4.1 |
| WHT.5R | <1 (equivalent) | 0.76 | 4.1 |
| **Females** (n=40) |  |  |  |
| BMI | 8 (weak support) | 0.51 | 5.5 |
| WC | 6 (plausible) | 0.53 | 5.3 |
| WHR | 27 (unsupported) | 0.21 | 6.9 |
| WHtR | 0 (best) | 0.60 | 5.0 |
| WHT.5R | 2 (plausible) | 0.57 | 5.1 |

AIC - Akaike’s Information Criterion; BMI - Body mass index; WC – waist circumference; WHR – waist-to-hip ratio; WHtR – waist-to-height ratio; WHT.5R – WC/height^0.5^

*The 95% confidence interval for the standard error of the estimate is ×/÷ a factor of 1.25 at these degrees of freedom.
