# Supplementary material for: Prediction of whole-body fat percentage and visceral adipose tissue mass from five anthropometric variables
Source: PLoS One. 2017 May 11;12(5):e0177175. doi: 10.1371/journal.pone.0177175 (PMC5426673; doi:10.1371/journal.pone.0177175)
Supplement: S3 Table — AIC—Akaike’s Information Criterion; BMI—Body mass index; WC—waist circumference; WHR—waist-to-hip ratio; WHtR—waist-to-height ratio; WHT.5R - WC/height0.5 *8 females excluded due to undetectable levels of VAT mass leading to badly behaved residuals. (DOCX) [file pone.0177175.s004.docx]

**Table 3: Prediction of VAT mass (Log) from anthropometric measures**

|  | AIC Difference  (Inference) | Adjusted R^2^ | Standard Error of the Estimate ×/÷ factor  (95% CI) |
| --- | --- | --- | --- |
| **Males** (n=41) |  |  |  |
| BMI | 13 (weak support) | 0.60 | 2.0 (1.8 to 2.4) |
| WC | 4 (plausible) | 0.68 | 1.8 (1.6 to 2.1) |
| WHR | 41 (unsupported) | 0.21 | 2.6 (2.2 to 3.3) |
| WHtR | 0 (best | 0.71 | 1.8 (1.6 to 2.1) |
| WHT.5R | <1 (equivalent) | 0.71 | 1.8 (1.6 to 2.1) |
| **Females** (n=32*) |  |  |  |
| BMI | 6 (plausible) | 0.58 | 2.8 (2.3 to 4.1) |
| WC | 3 (plausible) | 0.61 | 2.7 (2.2 to 3.7) |
| WHR | 25 (unsupported) | 0.22 | 4.1 (3.0 to 6.7) |
| WHtR | 0 (best) | 0.65 | 2.6 (2.2 to 3.7) |
| WHT.5R | <1 (equivalent) | 0.64 | 2.6 (2.2 to 3.7) |

AIC - Akaike’s Information Criterion; BMI - Body mass index; WC – waist circumference; WHR – waist-to-hip ratio; WHtR – waist-to-height ratio; WHT.5R - WC/height^0.5^

*8 females excluded due to undetectable levels of VAT mass leading to badly behaved residuals
